# Internal Overview of Prostatic Cancer Cases and Quality of *BRCA1* and *BRCA2* NGS Data from the FFPE Tissue

**DOI:** 10.3390/diagnostics14182067

**Published:** 2024-09-18

**Authors:** Enrica Antolini, Alessandra Filosa, Matteo Santoni, Elena Antaldi, Elisa Bartoli, Lidia Sierchio, Federica Giantomassi, Alessandra Mandolesi, Gaia Goteri

**Affiliations:** 1 Department of Biomedical Science and Public Health, Università Politecnica delle Marche, 60126 Ancona, Italy; enricaa.58@gmail.com (E.A.); f.giantomassi@virgilio.it (F.G.); g.goteri@univpm.it (G.G.); 2 Anatomic Pathology Unit, Azienda Ospedaliero Universitaria delle Marche, 60126 Ancona, Italy; elena.antaldi@ospedaliriuniti.marche.it (E.A.); elisa.bartoli@ospedaliriuniti.mache.it (E.B.); lidia.sierchio@ospedaliriuniti.marche.it (L.S.); alessandra.mandolesi@ospedaliriuniti.marche.it (A.M.); 3 Oncology Unit, Macerata Hospital, Via Santa Lucia 2, 62100 Macerata, Italy; matteo.santoni@sanita.marche.it

**Keywords:** prostate cancer, molecular biology, tumor biomarkers, PARPi, FFPE, sequencing, NGS, *BRCA1*, *BRCA2*, ctDNA, liquid biopsy

## Abstract

**Background:** Comprehensive genomic profiling (CGP) has gained an important role in patients with advanced prostate cancer following the introduction of PARP inhibitors in daily clinical practice. Here, we report an overview of CGP results, specifically of BRCA1 and BRCA2 HRD-repair system genes, from patients with prostate cancer analyzed in our institution, and we compare our results with those available from more recent scientific literature. **Methods:** The study cohort consisted of 70 patients. Somatic DNA was extracted from Formalin-Fixed Paraffin-Embedded (FFPE) tissue using a MagCore Genomic DNA FFPE One-Step Kit for MagCore System. The DNA was quantified by EasyPGX^®^ Real-Time qPCR and EasyPGX^®^ Analysis Software (version 4.0.13). Tissue somatic DNA libraries were prepared with Myriapod^®^ NGS BRCA1-2 panel-NG035 and sequenced in a Mi-Seq^®^ System. The sequence alignment in hg19 and the variant calling were performed using Myriapod^®^ NGS Data Analysis Software version 5.0.8 NG900-SW 5.0.8 with a software detection limit (LoD) of 95%. Variants with a coverage of 500 and VAF% ≥ 5 were evaluated. **Results:** Tumor tissue NGS was unsuccessful in 46/70 patients (66%). Mutations of the *BRCA2* gene were detected in 4 of the samples: (1) *BRCA2* ex10 c.1244A>G p.His415Arg VAF = 51.03%; (2) *BRCA2* ex11 c.5946delT p.Ser1982fs VAF = 72.1%; (3) *BRCA2* ex11 c.3302A>G p.His1101Arg VAF = 52.9%; and (4) *BRCA2* ex11 c.3195_3198delTAAT p.Asn1066fs VAF = 51.1%. **Conclusions:** The results from our internal overview seem to support the data and to confirm the performance of the technical issues reported in the literature. Considering the advanced age of our patients, with 84% of men over the age of 65, the application of alternative and less invasive procedures such as liquid biopsy, could be a more suitable solution for some cases.

## 1. Introduction

### 1.1. Prostate Cancer Diagnosis and Treatment

Prostate cancer has become the most frequent tumor in the male population of western countries [1]. Imaging technical improvements in more recent years favors diagnostic accuracy and facilitates a tailored therapy approach in prostate cancer patients. In Italy, prostate cancer represents 19.8% of all male tumors. A total of 80% of cancers at diagnosis are limited to the anatomical structure of the prostate gland and are surgically curable, with an estimated life expectancy of up to 99% over 10 years for localized PCa patients [1,2,3]. A minority of patients have local positive lymph nodes (about 15%) or distant metastasis (5%) at the diagnosis, with a reduction of survival rate to 5 years at 30–40% [3,4]. Although only 5% have an advanced stage [4], the incidence rate of such cases has risen [1]. The majority of patients develop a castration-resistant prostate cancer (CRPC) after a median duration of treatment of 18–48 months. The transition to a castrate resistance state could rely on alternative survival pathways, some related to androgen-independent mechanisms. Prostate cancer is also among one of the most heritable of human cancers, with the estimate of 57% (95% confidence interval [CI]) of the inter-individual variation in risk attributed to genetic factors [5]; hence, the importance of the study about somatic and germinal alterations and depletion of the tumor prostatic cell’s genome. Somatic genomic mutations contribute to cancer by altering the function of genes or compromising signal pathways important for tumorigenesis, metastasis, and therapy resistance mechanisms [6]. DNA-repair system gene alterations are involved in prostate cancer developments and are associated with metastatic therapy-resistant prostate cancer (mCRPC) [6,7]. Approximately 90% of mCRPC harbor clinically actionable, somatic or germline, gene alterations. Non-AR-related actionable alterations included aberrations in the PI3K pathway (49%), DNA repair pathway (19%), RAF kinases (3%), and CDK inhibitors (7%).

### 1.2. BRCA1 and BRCA2 Gene Mutations

A pivotal role in prostate cancer biology is played by the tumor suppressors *BRCA1* and *BRCA2*, belonging to the homologous recombination deficiency (HRD) repair system, that enables the error-free recovery of double strand breaks (DSBs) [8,9]. *BRCA1* is located on chromosome 11q21 and *BRCA2* on chromosome 13q12–13 [10,11,12]. More than 3500 mutations have been reported for *BRCA1* and *BRCA2*. It has been estimated that 0.1–0.2% of the population are carriers of those mutations [13,14].

Castro et al. have described and characterized *BRCA2* as an important prognostic factor for aggressive prostate cancer (PCa) [8]. Carriers of germline *BRCA2* pathogenic sequence variants have elevated aggressive prostate cancer risk and are candidates for precision oncology treatments [9]. Robinson et al. have identified mutations in three DNA repair genes, *BRCA1*, *BRCA2*, and *ATM*, in men unselected for age at diagnosis or family history, but rather for aggressive disease [7], and the 19.3% of prostate cancers and 23% of prostatic cancer castration-resistant patients had *BRCA1*, *BRCA2* or *ATM* mutations [7,13]. Several studies have focused on the classification with prognostic and therapeutic implications based on these driver mutations [14]. The prognostic significance of germline variants in homologous recombination repair genes in advanced prostate cancer (PCa), especially with regard to hormonal therapy, remains controversial.

### 1.3. PARP Inhibitors in Prostate Cancer Therapy

Tumor *BRCA* mutate cells are turned out sensitive to platinum and Poly (adenosine diphosphate [ADP-ribose] polymerase (PARP) inhibitor treatments [15]. PARP inhibitor (PARPi) are a class of anti-cancer drugs that compete with nicotinamide (NAD^+^) for the catalytically active site of PARP molecules. Inhibition of PARP activity was initially demonstrated in 1971 by Preiss ‘s group tested in HeLa cells [16]. PARPi have been shown to be effective against homologous recombination repair deficient tumor cells, with mutations that lead to loss of function of the proteins involved in the control system, but compatible with cell viability [17]. In 2005, it was originally hypothesized that loss of function of PARP1 molecular activity could have led to replication fork collapse, an event requiring a homologous recombination (HR)-dependent repair of these forks. In *BRCA1* and *BRCA2* mutated tumor cells, the HRD activity was compromised; therefore, the collapsed replication forks are unable to be repaired, and cell death occurs [18,19]. Today, PARP-inhibitors are currently used for the treatment of prostate cancer patients with germinal or somatic mutations in *BRCA1* and *BRCA2* genes, which results in therapeutically resistant cancers [20,21].

Additional FDA approvals have expanded the use of PARP inhibitors to more situations. People with a PALB2 mutation who have been diagnosed with castration resistant prostate cancer could be eligible for PARP inhibitors therapy; although, in the largest study of people with inherited PALB2 mutations, the gene was linked to increased lifetime risk of breast cancer in women and men, ovarian and pancreatic cancer but not prostate cancer [22,23,24].

### 1.4. NGS Technology Application in Prostate Cancer

Next generation sequencing (NGS) represents the most important technology to obtain the molecular information about these genes and other specific biomarkers in order to choose the best therapeutic approach and to better evaluate prognosis in prostate cancer patients. Comprehensive multigene genetic testing is necessary for appropriate clinical management of the pathogenic variants’ carriers. Additionally, the information obtained is important for determining the risk of malignancy development in family members of the examined individuals.

On the basis of the most relevant literature results, we describe an overview of our NGS *BRCA1* and *BRCA2* data of prostate cancer patients’ FFPE samples, and we highlight technical procedures performed and relevant clinical implications. Our aim was to compare our technical approach to those described in the literature, in order to find the most efficient procedure useful for detecting somatic mutation implicated in both tumorigenesis and therapy resistance. Moreover, we aimed to select the less invasive procedure with the best performance in order to ensure the best sequencing quality and to avoid a high percentage of not assessable data.

## 2. Materials and Methods

### 2.1. Patients and Tissue Samples

A cohort of 70 patients with a histological diagnosis of metastatic and non-metastatic prostate cancer was analyzed in this work. They were males, aged between 49 and 90 years, and referred to the Pathological Anatomy Unit of Azienda Ospedaliera e Universitaria delle Marche. We considered as the main inclusion criteria a prostate cancer diagnosis with a subsequent NGS analysis after proper oncological evaluation and indication.

Formalin-Fixed Paraffin-Embedded (FFPE) tissue was recovered for each patient between 2022 to 2023. Samples were fixed in a formalin neutral buffer for 12–24 h based on specimen size. The types of samples recruited were prostatic biopsies, prostatic surgical resections, and samples of metastatic locations. The specimens were considered suitable for sequencing if the neoplastic cells represented 50% of the tissue or if more than 100 neoplastic cells were observed in the slide. A pathologist selected the most representative cancer area on the slides and the technicians obtained the corresponding section from the selected block, for sequencing.

### 2.2. Somatic DNA Extraction and Sequencing of BRCA1 and BRCA2 Genes

Somatic DNA was extracted from the Formalin-Fixed Paraffin-Embedded (FFPE) tissue using a MagCore Genomic DNA FFPE One-Step Kit for MagCore System (Diatech Pharmacogenetics s.r.l., Ancona, Italy), following the manufacturer’s instructions, or manual extraction with a QIAamp DNA Mini kit (QIAGEN GmbH, QIAGEN Strasse 1, 40724 Hilden, GERMANY) to obtain a good extraction for a smaller sample size. The DNA was quantified in Real-Time qPCR by EasyPGX^®^ qPCR instrument 96 (Diatech Pharmacogenetics s.r.l., Ancona, Italy) and EasyPGX^®^ Analysis Software version 4.0.13 (Diatech Pharmacogenetics s.r.l., Ancona, Italy). Samples of DNA with a concentration of 0.4–2 ng/µL and fragmentation index >0.3 were selected for sequencing.

Tissue somatic DNA libraries were prepared with Myriapod^®^ NGS BRCA1-2 panel- NG035 (Diatech Pharmacogenetics s.r.l., Ancona, Italy), and Pool Library was quantified by Qubit^®^ dsDNA HS Assay Kits on Qubit 3.0 fluorometer (Invitrogen, Carlsbad, CA, USA) and sequenced in platform Mi-Seq^®^ System (Illumina, San Diego, CA, USA). The sequence alignment in hg19 and the variant calling were performed using Myriapod^®^ NGS Data Analysis Software version 5.0.8 NG900-SW 5.0.8 (Diatech Pharmacogenetics s.r.l., Ancona, Italy) with a software detection limit (LoD) of 95%. Variants with coverage 500 and VAF% ≥ 5 were evaluated. Variants with coverage of less than 500, VAF% loss of 5, and rare variants not reported as pathogenetic were discarded.

## 3. Results

The study population consisted of 70 male subjects aged between 49 and 90 years, all Caucasians and all with a diagnosis of prostatic adenocarcinoma with different stages. Of these, 59 had prostatic localization (stage pT2) and 11 (7.7%) were in advanced metastatic stage (Table 1) with lymph node, femoral, hepatic, pulmonary, vertebral, cavernous body, and vesical localizations. Furthermore, Table 1 shows the distribution of the prostate cancer Grade Group (WHO 2022) of 22 cases of our study population: 7 are placed in Grade Group 4 and 7 in Grade Group 5, 5 are placed in Grade Group 3, and 3 in Grade Group 1. The subjects with a known therapy totaled 5, three of which were treated with anti-androgen therapy, and 2 in therapy with Dutasteride (Table 1).

*BRCA1* and *BRCA2* sequencing data obtained from tissue somatic DNA of prostate cancer patients were analyzed. Among the 70 cases, 46 sequencing data could not be evaluated due to the poor quality and high fragmentation of the DNA obtained (fragmentation index <0.3).

The DNA of these cases was respectively extracted from 30 prostate biopsies, 11 prostate surgical resections, 1 clot included lymph node, 1 lung needle biopsy, 1 cavernous body needle biopsy, 1 femoral, and 1 liver biopsies. A total of 20 of the analyzed samples resulted in wild type for *BRCA1* and *BRCA2* genes, extracted respectively from 13 prostate biopsies, 2 prostate surgical resections, 2 clots included in mediastinal lymph node, 2 bladder biopsies, and 1 vertebral needle biopsy. Finally, mutations of *BRCA2* gene were detected in 4 of the samples: (1) *BRCA2* ex10 c.1244A>G p.His415Arg VAF = 51.03%, classified like Uncertain in NCBI (prostate surgical resection); (2) *BRCA2* ex11 c.5946delT p.Ser1982fs VAF = 72.1%, classified like Pathogenic in NCBI (prostate biopsy); (3) *BRCA2* ex11 c.3302A>G p.His1101Arg VAF = 52.9%, classified like Uncertain in NCBI (clots included mediastinal lymph node); and (4) *BRCA2* ex11 c.3195_3198delTAAT p.Asn1066fs VAF = 51.1 %, classified like Pathogenic in NCBI (prostate biopsy) (Table 2).

## 4. Discussion

The investigation of inherited and acquired defects in DNA damage repair system are the key for understanding the underlying mechanisms of the genesis of tumors. The detection of mutations in DNA-repair genes allows the identification of cancer familiar predisposition and the definition of cancer subtypes that are sensitive to specific therapeutic treatments [22].

### 4.1. Importance of BRCA1 and BRCA2 Genes

**Figure 1:**
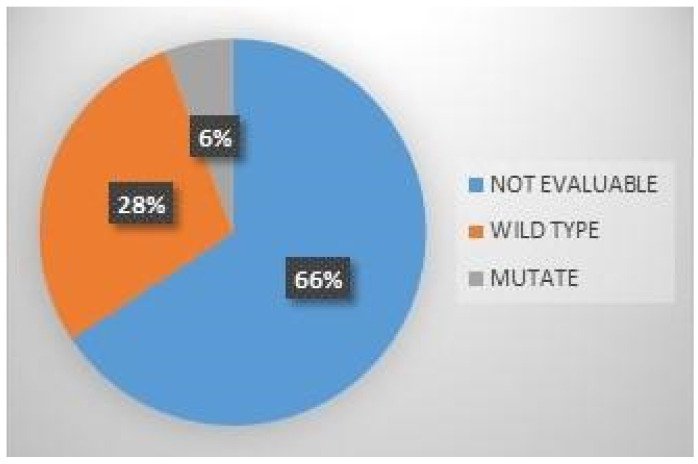
Percentage results in not evaluable, wild type, and mutate; obtained from *BRCA1* and *BRCA2* NGS sequencing of the 70 cases of the study population.

After recognizing the role that *BRCA1* and *BRCA2* genes have in susceptibility and prognostic evaluation in breast and ovarian cancer [10,11], several works have studied the association between aberrations of DNA-repair system genes and prostate cancer [13,22,23,24]. Loss-of-function alterations in these genes causes genomic instability leading to the transformation of the tumor cells but, at the same time, can be exploited for therapeutic strategies [25,26,27]. In fact, *BRCA1* and *BRCA2* mutated cells are more responsive to anti-tumor treatments that induce the accumulation of damage to the DNA double strand, the so-called PARP-inhibitor [15]. From data of the multicenter study TOPARP-B, it emerged that the 53.4% of castration-resistant prostate cancer patients and DNA repair mutated, treated with Olaparib, had a composite response at two years of follow-up [28]. From the GALAHAD study, which has investigated treatments of castration-resistant prostatic carcinoma with Niraparib, it emerged that 65% of patients’ diagnoses with a *BRCA1/2*-mutated prostate carcinoma and 31% of patients with alternative DNA-repair gene mutated prostate cancer achieved a composite response [28]. In subjects with metastatic-resistant prostate cancer, the effectiveness of treatment with Olaparib has been demonstrated [29,30]. *BRCA2* is the most frequently mutated gene among the DNA-repair system in subjects with prostate cancer, with a frequency of somatic alterations of 8–12%, of which the germline is 5.3%. Alterations of *BRCA1* genes are less frequent; 0.9% in somatic DNA, of which the germline is <0.3% [31,32,33]. Our data of 6% of the *BRCA2* mutated samples is close to the frequency generally found; this, despite the small number of subjects in our population and the high rate of not evaluable data (Figure 1).

### 4.2. Challenges of NGS Technology

The advent of next generation sequencing (NGS) has made it possible to obtain much more molecular information form the FFPE tissue [34]. However, the DNA obtained from FFPE tissue is often of low quality for the available sequencing systems. Although formalin can preserve the morphology and properties of tissue, several works have underlined the problems arising from formaldehyde-protein-nucleic acid interaction [34].

Formalin fixation is the most critical step in the pre-analytical phase of oncological tissue sample. An adequate fixation time is of pivotal importance in order to preserve, as much as possible, the integrity of nucleic acids. Around 12–24 h fixation in neutral buffered formalin is usually recommended to obtain a good sample preservation for a morphological evaluation. A shorter time may lead to incomplete fixation, which determines the enzymatic degradation of the tissue, while a longer time may cause more extensive cross-linking and a more difficult extraction of DNA and RNA [35,36,37]. In fact, the DNA and RNA extraction yield may decrease with increasing storage time [38].

Formaldehyde can seriously damage the DNA double helix, and can create a cross-link with the structure of DNA like histone-DNA cross-links, DNA-protein cross-licks, formaldehyde-DNA adducts, and DNA-DNA cross-links [31,34,38,39,40]. Moreover, DNA from FFPE is more subject to a deamination of cytosine and 5′ methyl-Cytosine, forming uracil and thiamine. These can also cause sequence artifact by the DNA polymerase, like C:G>T:A, C:G>A:T, C:G>G:C, and A:T>G:C, or else an a-basic site may form, which can alter the structure of the DNA double helix or may beaks it [41]. Therefore, formaldehyde in FFPE tissue can be considered the principal cause of 66% of our not evaluable data, coming from a highly fragmented DNA (Figure 1). Since the first works regarding the quality of the FFPE-derived DNA for NGS-based analyses were published [42], more effective protocols on the use of formalin have been implemented within the NGS pipeline. In most of them, time between specimen removal and fixation are more controlled and evaluated in relation to the size of the tissue sample [43,44,45,46].

### 4.3. Practical Implications

The high percentage of not evaluable data in our study could be due to formalin-fixed tissue fragmentation. This, in particular, in less recent cases for which a standardization protocol of fixation was not available. For these cases, at the time of diagnosis, the possibility of a target therapy with PARP inhibitors was not predictable. These results combined with the small series described, limit the clinical value of our study. Therefore, our aim was merely that of suggesting an alternative method, available in other cancer patients, to avoid our limitations.

The current clinical diagnostic approach is histologic diagnosis followed by DNA analysis from prostate biopsy specimen in FFPE. For the tumor comprehensive genomic profiling (CGP), circulating tumor cell-free DNA analysis (ctDNA), a small fraction of circulating cell-free DNA (cfDNA) may prove to be a better choice. In order to obtain information about alterations of prostate cancer biomarkers, ctDNA and ctRNA, obtained by serum and urine, are representative. The information obtained from ctDNA sequencing provides an indication regarding the nature of the primary lesion and metastases, and encourages the comprehension of the tumor landscape; furthermore, it is a less invasive and easily repeatable procedure [47,48,49,50,51,52,53].

## 5. Conclusions

The results obtained from our case series support the data and technical issues reported in the literature. The importance of providing molecular information on the *BRCA1* and *BRCA2* genes is widely recognized, especially for patients with metastatic prostate cancer. The effectiveness of PARPi therapies for resistant prostate cancer with alterations in DNA-repair genes, in particular *BRCA1* and *BRCA2*, has been widely tested. The America guidelines of The National Comprehensive Cancer Network (NCCN) recommend germline and/or somatic NGS sequencing of homologous recombination repair (HHR) genes (*BRCA1*, *BRCA2*, *ATM*, *CHEK2, PALB2*, *MSH2*, *MSH6,* and *PMS2*) or *BRCA* testing to identify pathogenic mutations for the treatment with PARPi (Olaparib and Rucaparb) [54,55].

Considering the advanced age of the patients (84% of subjects in our study are over the age of 65) and the difficulties encountered for the extraction of a good quality DNA from FFPE tissue samples, the application of alternative and less invasive procedures, like liquid biopsy, could be a solution for specific cases. The analysis of ctDNA obtained from our patients’ plasma sample would provide a timely result useful to the therapeutic choice and easily reproducible for prognostic monitoring, thus offering also the possibility of testing germline mutation.

## Figures and Tables

**Table 1:** Study population: average age, stage and classification of the prostatic cancer, therapeutic treatments.

Average Age	Primary Prostatic Adenocarcinoma	Metatatic Prostatic Adenocarcinoma	Grade Group 1 *	Grade Group 2 *	Grade Group 3 *	Grade Group 4 *	Grade Group 5 *	Therapy *
49–90	59	11	3	/	5	7	7	3: anti-androgen therapy 2: therapy with Dutasteride

* We have reported the available information. Since part of our samples come from consultations, it was not possible to obtain complete clinical and therapeutic information of all the patients.

**Table 2:** Analysis results of the sequencing data of *BRCA1* and *BRCA2* gene from tissue somatic DNA of the 70 prostate patients of this work.

Sample Type	Not Evaluable	Wild Type	Mutate
Prostate biopsy	30	13	-*BRCA2* ex11 c.5946delT p.Ser1982fs VAF = 72.1 %, Pathogenic -*BRCA2* ex11 c.3195_3198delTAAT p.Asn1066fs VAF = 51.1 %, Pathogenic
Prostate surgical resection	11	2	*BRCA2* ex10 c.1244A>G p.His415Arg VAF = 51.03, Uncertain
Cot included—Supraclavicular lymph nodes			*BRCA2* ex11 c.3302A>G p.His1101Arg VAF = 52.9 %, Uncertain
Cot included—Mediastinal lymph nodes		2	
Cot included—Lymph nodes	1		
Lung needle biopsy	1		
Femoral biopsy	1		
Bladder biopsy		2	
Vertebral needle biopsy		1	
Cavernous body needle biopsy	1		
Liver biopsy	1		

## Data Availability

The information is contained within this article in its entirety. For additional information, please feel free to inquire with either the original author or the corresponding author. Public access to the data is restricted as a result of the patient privacy standards that regulate the handling of clinical data.
